# Non-operative management of a rare diagnosis of splenic torsion in a child with a history of giant omphalocele: a case report and literature review

**DOI:** 10.1186/1754-9493-8-12

**Published:** 2014-03-07

**Authors:** Fariha Sheikh, Michael E Kim, Irving J Zamora, Oluyinka O Olutoye

**Affiliations:** 1 Division of Pediatric Surgery, Michael E DeBakey Department of Surgery, Baylor College of Medicine and Texas Children’s Hospital, Houston TX, USA; 2 Pediatrics and Obstetrics & Gynecology, Baylor College of Medicine, Texas Children’s Hospital, 6701 Fannin St. Suite 1210, Houston, TX 77030, USA

**Keywords:** Splenic torsion, Omphalocele, Non-operative management, Wandering spleen

## Abstract

**Background:**

Splenic torsion is rare and as a result the appropriate management is unclear. While there has been a shift towards splenectomy and laparoscopic splenopexy, we present a successful case of non-operative management of splenic torsion in a patient with a history of a giant omphalocele.

**Case presentation:**

A 3 year-old female presented with a three-day history of abdominal pain, fever and non-bloody emesis three and a half years after repair of her giant omphalocele. Abdominal radiographs and ultrasound demonstrated migration of the spleen and a subsequent computerized tomography scan confirmed splenic torsion and an infarcted spleen. Given her late presentation, she was successfully managed with observation, analgesia, immunization against capsulated organisms and daily penicillin prophylaxis with excellent outcome at 19 months follow-up. A review of the literature revealed that splenic torsion is rarely managed non-operatively. Rarer still is the occurrence of splenic torsion following a history of omphalocele.

**Conclusion:**

Although rare, splenic torsion should be considered in a child with a history of omphalocele presenting with abdominal pain. Non-operative management of an infarcted spleen can be a safe treatment option to avoid surgery in complex patients.

## Background

Splenic torsion and wandering spleen are rare causes of abdominal pain. The splenic attachments, when absent or underdeveloped, permit the spleen to migrate from its typical position in the left upper quadrant of the abdomen to anywhere in the abdomen [1]. The wandering, mobile spleen can then twist around its vasculature resulting in possible gangrene, hemorrhagic infarct, or splenic pseudocyst [2,3]. Adult females of childbearing age and children under the age of ten years exhibit the highest presentation of wandering spleens [4-6]. The overall incidence of splenic torsion or wandering spleen is variable with 486 cases reported worldwide, in the English literature [5-7]. Within the pediatric population the etiology is typically due to malformation of the splenic attachments including the gastrosplenic, splenorenal, splenophrenic, splenocolic, splenopancreatic, presplenic fold, pancreaticocolic, and phrenocolic ligaments [7].

Splenic torsion is difficult to diagnose due to presentation with symptoms that are non-specific including abdominal pain, fever, nausea and vomiting. Symptoms may or may not be accompanied by palpation of an abdominal mass on physical exam. Thus, a high index of suspicion and imaging is required for accurate diagnosis. Sonography is the imaging study of choice for wandering spleen although CT scan may be needed in certain cases to confirm diagnoses [1-3]. In depth knowledge of the patient’s medical history may heighten suspicion for this diagnosis, as this may occur in patients at risk for malrotation such as those with large omphalocele or congenital diaphragmatic hernia [3,4]. Here we present a pediatric patient who previously underwent repair of a large omphalocele, and presented with splenic torsion that was managed non-operatively. Following this case we performed a review of the literature on the treatment of pediatric patients with splenic torsion and wandering spleen.

## Case presentation

**Figure 1:**
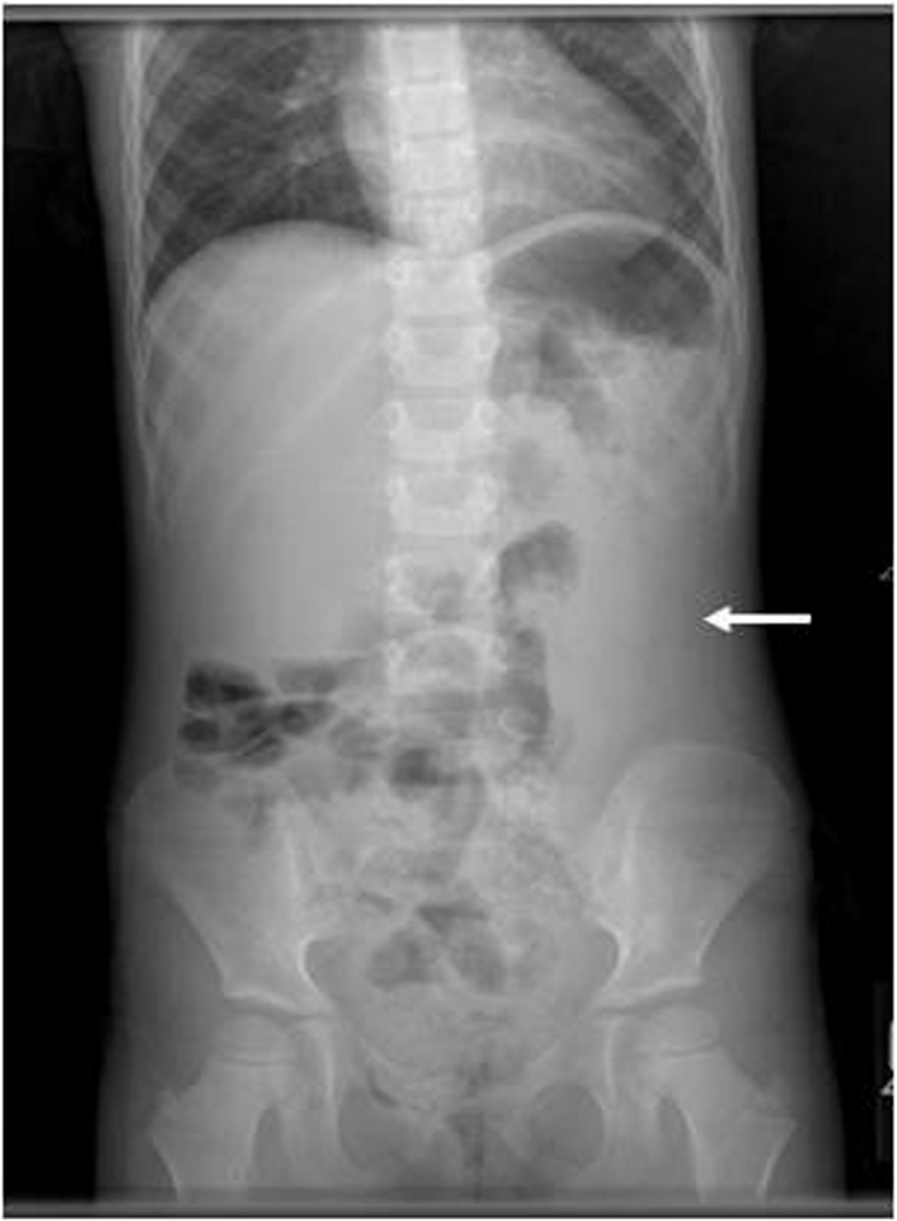
Plain radiograph of abdomen: mass effect noted in left mid-abdomen.

**Figure 2:**
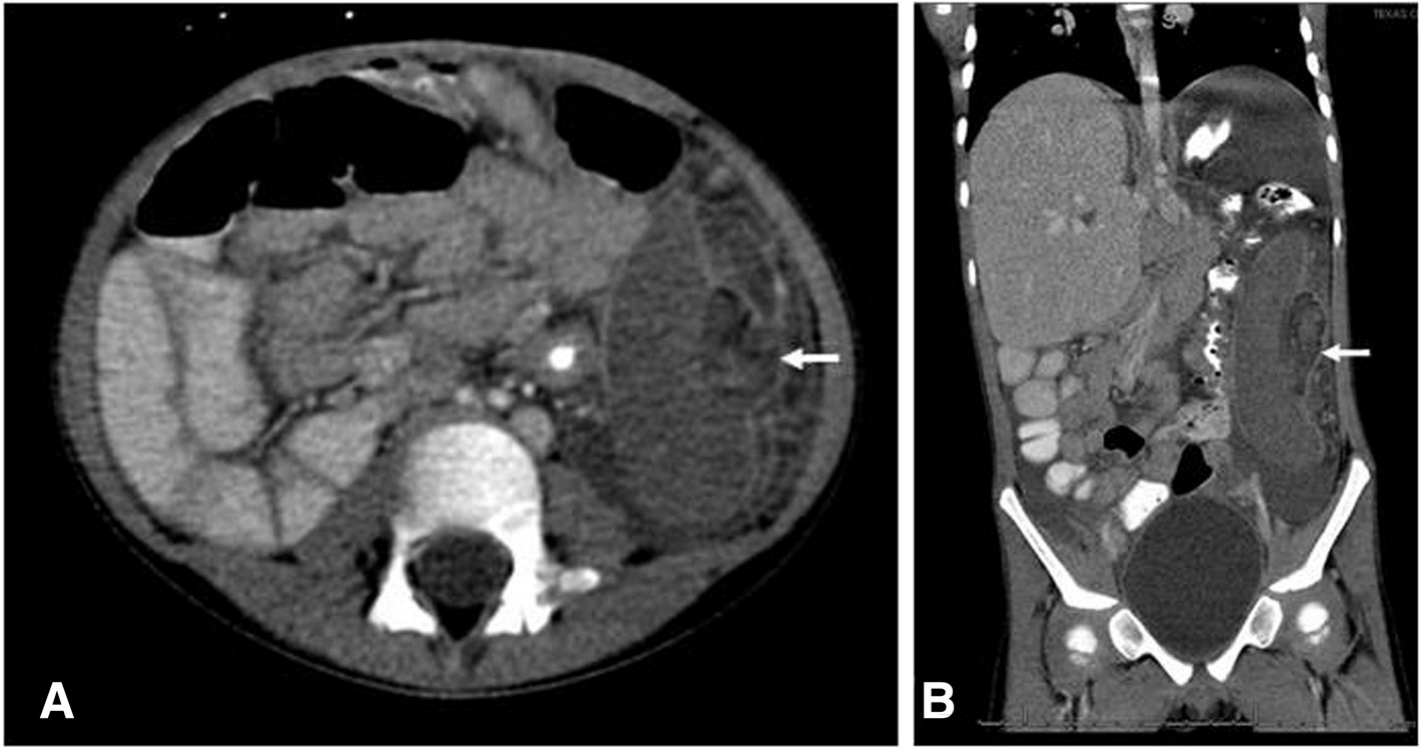
**CT imaging of infarcted spleen: (A)Axial image showing global infarction.(B)** Coronal view of CT scan of abdomen and pelvis with enteral and intravenous contrast. There is lack of contrast enhancement of the spleen with the hilum rotated externally (arrow).

A 41 month-old female with a history of prenatally diagnosed giant omphalocele that was repaired at nine months of age following epithelialization of the sac and gradual reduction of the omphalocele. She presented to the emergency room with a three-day history of left upper quadrant abdominal pain, fatigue, one episode of non-bloody, non-bilious emesis and fever to 102 F. Physical examination noted a well-healed abdominal midline scar but did not elicit abdominal tenderness or palpable mass. Abdominal plain film was significant for a mass within the left flank (Figure 1). White blood cell count was elevated to 17.54 10^3^ uL, the hematocrit was 31.2 and platelet count was 279 10^3^ uL. A CT scan showed the spleen to be located in the left flank, vertically oriented with the hilum facing laterally (Figure 2). There was loss of all parenchymal architecture and a thin rim of capsular enhancement, indicative of global infarction. A thickened and swirled appearance of the splenic hilum was observed with a cuff of fat surrounding the poorly enhancing splenic vessels, consistent with a torsed splenic pedicle. Significant edema was also found around the splenic region. The previously known intestinal malrotation had not changed. The supra hepatic inferior vena cava was markedly displaced anteriorly as is typically noted in with children a history of large omphaloceles. Due to the delayed presentation (3 days following onset of symptoms) and imaging features suggestive of global infarction of the spleen, we discussed the management options with the child’s parents and opted to proceed with non-operative management. Supportive care was provided until the abdominal pain resolved. She was immunized against encapsulated organisms and placed on daily prophylactic penicillin. She was tolerating a regular diet without fever or emesis by the time she was discharged home on hospital day 5. The family was counseled about the risks of overwhelming sepsis in the absence of a functioning spleen and to return for any signs of abdominal pain or worsening symptoms. She was seen in the emergency department 16 days following discharge complaining of abdominal pain but was afebrile. Ultrasonography was consistent with previous findings except the spleen size decreased from 11.3 cm to 9.4 cm. She was discharged home without subsequent hospital admissions. At 19 months follow up the patient reports no further abdominal pain and has tolerated a regular diet. She has maintained penicillin antibiotic prophylaxis without episodes of overwhelming post-splenectomy infections. She has been able to avoid surgery with no adverse effect.

## Discussion

There are six cases of wandering spleen reported in patients with a history of congenital diaphragmatic hernia. The literature consists of two prior reports of wandering spleen in patients with omphalocele; the first in 1987 and the second in 2008 which was managed by de-torsion of the spleen and splenopexy [8,9].

Management of splenic torsion has evolved from primarily a splenectomy approach towards increasing numbers of splenopexies via the laparoscopic approach [10]. Non-operative management has not been favored or discussed in the literature since 1996 when the risk of sepsis and a mortality rate of 30 to 60% was noted for those patients that did not undergo surgical treatment [3]. Surgical management is the current gold standard for treatment of splenic torsion. In the face of acute torsion, immediate surgery to detorse the spleen may result in splenic salvage. This can be performed laparoscopically or via an open approach. Splenopexy may also be considered at that time. Once the spleen is already infarcted, non-operative management may be considered as an alternative to splenectomy in this setting.

Prior to our case, reports in literature have not advocated non-operative treatment as a feasible plan of care complication rates as high as 65%, resulting in splenic rupture and infarction due to torsion of the splenic pedicle [2,3,5-7]. Our experience with this case allows us to present a non-operative approach that showed favorable outcomes. Contrary to previous reports, this patient did not experience a splenic rupture as a consequence of non-operative management for splenic torsion. Additionally, the spleen had already undergone complete infarction prior to her admission followed by an acute resolution of symptoms. We were able to monitor this patient through the period of infarction and were aware that she was not developing signs of sepsis which allowed for safe non-operative management, nor did she develop an overwhelming post-splenectomy infection following discharge. The special circumstances for this patient’s presentation prompted our decision to manage her non-operatively. We reviewed the literature for all pediatric cases of splenic torsion and the treatment since the last literature review published in 2003.

The initial method to diagnosing splenic torsion is to image the abdomen using either ultrasound or CT imaging, both sufficient methods used to diagnose splenic torsion and evaluate the vascular supply to the spleen [5,7]. Sonography has been cited in literature as a precise method for diagnosis and is particularly useful in children [2,3,5]. Magnetic resonance imaging has also been used but is rarely required to make the diagnosis [11]. The advantage of utilizing CT imaging involves evaluation of the vascular supply to the spleen, thus aiding in choosing between splenectomy, splenopexy, or non-operative management. Physical examination is not diagnostic due to the lack of definitive symptoms and signs, although in some cases an enlarged spleen may be palpated within the abdomen and can be distinguished by its mobility [2]. Delayed presentation in our patient with an already infarcted spleen allowed for careful observation, non-operative management, and prophylaxis for asplenia which resulted in a favorable outcome. CT scan imaging provided the advantage of visualizing complete splenic infarction, thus allowing non-operative treatment consisting of observation, administration of immunizations against encapsulated organisms, and prophylactic antibiotics without incurring adverse effects.

While surgical options are usually regarded as the most favorable course for treating splenic torsion, non-operative management can be a viable option. As demonstrated with our case, the patient had clearly developed an infarcted spleen at the time of CT scan but was exhibiting a stable clinical picture given the resolution of fever, stable vital signs and resolution of abdominal pain post emesis. The patient has since remained free of infection by maintaining a regimen of antibiotic prophylaxis and immunizations. The benefit is that she was able to avoid an unnecessary operation. Non-operative management allowed the course of splenic infarction to continue naturally and resemble autosplenectomy as seen in children with sickle cell anemia. Though not identical to splenic torsion, the same risks are present for both etiologies including infection and hemorrhage. While there has not been any literature focused on non-operative management of splenic torsion in over 10 years, there was a recent study that reviewed the management of 190 patients with acute splenic sequestration crisis from 2000 to 2009. The study demonstrated that the 103 that underwent watchful waiting management had a favorable outcome overall despite some patients having a recurrence of acute splenic sequestration crisis [12].

A literature review published in 2003 revealed 17 open splenectomy, 21 open splenopexy, and 2 laparoscopic splenopexy approaches for the management of splenic torsion and wandering spleen [13]. Since 2003 a total of 44 pediatric cases of wandering spleen have been reported worldwide in both the surgical and radiology literature. Of these cases, 40 included details on the operative management, the majority being splenopexy. One patient was operatively managed with an open partial splenectomy to remove only the infarcted area of splenic tissue followed by splenopexy for the viable portion of the spleen [14]. Within the patients who underwent splenopexy, three patients underwent splenopexy and gastropexy for gastric volvulus (two were performed by laparotomy and one by laparoscopy) [15-17]. Splenic cysts were noted in two cases thus requiring splenopexy and cystectomy, both performed as open procedures [18,19]. For patients that had viable spleens intraoperatively and underwent splenopexy, six had mesh placed to secure the spleen (four used vicryl mesh, one reported use of non-absorbable mesh, and one had polypropylene) [14,19-22]. The remainder of splenopexies used adjacent tissue from the diaphragm, anterior abdominal wall, or peritoneum to create pouches for the spleen. A pre-peritoneal distension balloon was utilized to create an extraperitoneal pocket for the spleen in one case which followed with an uneventful hospital course [17]. All of the published cases reported an uneventful course, though follow-up time varied.

A case published in 2003 reported an incidence of splenic rupture secondary to splenic torsion in a 25 year-old female. She was hypotensive, underwent an exploratory laparotomy and splenectomy and was placed on standard post-splenectomy precautions [23]. While this example makes the case for immediate surgical management of splenic torsion, our patient did not present in a septic state. Had our patient presented in a similar manner, operative management would have been the likely choice, however she maintained stable vital signs during admission and this guided the option of observation.

## Conclusion

We suggest that non-operative management for splenic torsion be considered a safe alternative approach to managing select patients that are in stable condition. While splenectomy and splenopexy have been the foundation for treatment and the increase in use of laparoscopy and mesh have offered innovative approaches with sound results, non-operative management may be considered as an additional option for patients that are high risk surgical candidates.

## Consent

Written informed consent was obtained from the patient’s parent for publication of this Case report and any accompanying images. A copy of the written consent is available for review by the Editor-in-Chief of this journal.

## Competing interests

The authors declare that they have no competing interests.

## Authors’ contributions

FS made substantial contributions to acquisition of data, drafting the manuscript, agreed to be accountable for all aspects of the work in ensuring that questions related to the accuracy or integrity of any part of the work are appropriately investigated and resolved. ME made substantial contributions to acquisition of data, drafting the manuscript, agreed to be accountable for all aspects of the work in ensuring that questions related to the accuracy or integrity of any part of the work are appropriately investigated and resolved. IZ has been involved in drafting the manuscript and agreed to be accountable for all aspects of the work in ensuring that questions related to the accuracy or integrity of any part of the work are appropriately investigated and resolved. OO has been involved in drafting the manuscript, revising it critically for important intellectual content, has given final approval of the version to be published, and agreed to be accountable for all aspects of the work in ensuring that questions related to the accuracy or integrity of any part of the work are appropriately investigated and resolved. All authors read and approved the final manuscript.

## Authors’ information

FS is a surgical resident and is currently completing a pediatric surgery research fellowship at Baylor College of Medicine.

MS has a master’s degree in anatomy, completed a research fellowship at Baylor College of Medicine and is a medical student at Midwestern University.

IZ is a surgical resident, currently completing a pediatric surgery research fellowship at Baylor College of Medicine, and obtaining his Masters in Public Health.

OO is an attending pediatric surgeon, has a PhD in anatomy and is a professor of surgery, pediatrics, and obstetrics and gynecology at Baylor College of Medicine.
